# Metabolomic Profiling of Hormonal Contraceptive Use in Young Females Using a Commercially Available LC-MS/MS Kit

**DOI:** 10.3390/metabo13101092

**Published:** 2023-10-18

**Authors:** Tania Grobler, Monique Opperman, Janette Bester, Albe Carina Swanepoel, Ilse du Preez

**Affiliations:** 1 Centre for Human Metabolomics, North-West University, Potchefstroom 2531, South Africa; 2 Department of Physiology, Faculty of Health Sciences, School of Medicine, University of Pretoria, Pretoria 0002, South Africa

**Keywords:** drospirenone, estradiol, hormonal contraceptives, metabolic profiling

## Abstract

Oral hormonal contraceptive users carry the risk of venous thrombosis and increased mortality. This study aimed to comprehensively profile the serum metabolome of participants using a combination of drospirenone (DRSP) and ethinyl estradiol (EE) containing oral contraceptives (COCs). The MxP Quant 500 kit for liquid chromatography mass tandem spectrometry (LC-MS/MS) was used to analyse the 22 controls and 44 COC users (22 on a low EE dose (DRSP/20EE) and 22 on a higher EE dose (DRSP/30EE)). The kit’s results were compared to our internally developed untargeted and targeted metabolomics methods previously applied to this cohort. Of the 630 metabolites included in the method, 277 provided desirable results (consistently detected above their detection limits), and of these, 5 had *p*-values < 0.05, including betaine, glutamine, cortisol, glycine, and choline. Notably, these variations were observed between the control and COC groups, rather than among the two COC groups. Partial least squares-discriminant analysis revealed 49 compounds with VIP values ≥ 1, including amino acids and their derivatives, ceramides, phosphatidylcholines, and triglycerides, among others. Ten differential compounds were consistent with our previous studies, reinforcing the notion of COCs inducing a prothrombotic state and increased oxidative stress. Although only a limited number of compounds were deemed usable, these were quantified with high reliability and facilitated the identification of meaningful biological differences among the sample groups. In addition to substantiating known drug-induced variations, new hypotheses were also generated.

## 1. Introduction

Oral hormonal contraceptives (OHCs) are used by more than 151 million females globally [1] and form a fundamental part of modern health. Despite being listed as an essential medicine by the World Health Organization [2], OHC use, particularly that of combined oral contraceptives (COCs), is an important risk factor for venous thrombosis (VT), consisting of pulmonary embolism and deep vein thrombosis (DVT), which, in turn, is associated with increased mortality risk [3].

Currently, the omics approach encompassing genomic [4], transcriptomic [5], proteomic [6], and metabolomic analyses [7] can resolve many questions related to complex molecular processes while providing valuable clinical datasets. Pharmacometabolomics, a subfield of metabolomics, also referred to as pharmacometabonomics, evaluates the effect of drug exposure on a cohort or individual’s metabolome profile [8]. By applying an untargeted, two-dimensional gas chromatography time-of-flight mass spectrometry (GCxGC-TOFMS) pharmacometabolomics approach, we previously exploratively investigated the effect of COCs containing drospirenone (DRSP) and ethinyl estradiol (EE) on the serum metabolome profiles of females, as a means to identify underlying mechanisms of the drug-induced prothrombotic state [9].

Here, a total of 848 features were detected and, of the 255 features which were significantly different between the COC user groups and controls, the majority were annotated as amino acids using commercial library matches. The identified metabolic variations were linked to specific biophysical coagulatory variations previously described in COC use.

To build on these results, we then applied a targeted approach, normally used to diagnose inborn errors of the metabolism, to absolutely identify and quantify a subset of amino acids and acylcarnitines using compound standards and isotopes [10]. In this case, the metabolome variations which could be associated with COC use corresponded to those detected in our preceding untargeted study and correlated to amino acid variations detected by other groups, for earlier-generation COCs. Although disparities existed between the acylcarnitine profiles of the sample groups, no statistically significant variations were identified. Amino acids and acylcarnitines are, however, only a small subset of the human metabolism.

To obtain a more holistic view of the metabolome variations, in the current study, we aimed to comprehensively profile the serum of these cohorts by using a commercially available kit. This kit is said to be able to quantify over 600 compounds from 26 biochemical classes, in a targeted manner, using liquid chromatography mass tandem spectrometry (LC-MS/MS) analysis, after a relatively simple sample preparation step. The outcomes were then compared to those obtained with our in-house-developed untargeted and targeted (amino acid and acylcarnitine) methods, applied previously.

## 2. Materials and Methods

### 2.1. Cohort Recruitment and Sample Collection

Participants were actively recruited for this study, and the cohort included young adult females (18 to 30 years of age) who were non-smokers, had no known chronic conditions (including thrombotic diseases), and, apart from the relevant COCs, were not taking any chronic medication at the time of sample collection. Participants were grouped into three categories, namely a control group (females who have not used any hormonal contraceptives for 6 months prior to sample collection), DRSP/20EE users (females using a 3 mg DRSP and 20 µg EE combination in each active tablet), and DRSP/30EE users (females using a 3 mg DRPS and 30 µg EE combination in each active tablet). To minimize the impact of endogenous female sex hormones, blood was collected from the control group during menses [11]. Samples were collected from the COC users if the active pills were ingested for at least seven consecutive days, which reflects the time it takes for the exogenous hormones to stabilize [12]. At this point, the synthetic hormones will ‘override’ the action of endogenous hormones, since the exogenous progestin concentration is up to eightfold higher than the median value of endogenous progesterone throughout the 28-day cycle in non-COC users, and the exogenous estrogen is equal to the maximum concentration of the endogenous estradiol during the cycle [13].

A qualified phlebotomist drew venous blood from each of the participants directly into a VACUCARE plain red tube (no additives). For the purpose of clot formation, the collected samples were left to stand for 30–60 min at room temperature, whereafter they were centrifuged at 4 °C for 5 min at 3000 rpm. By using a glass Pasteur pipette, the supernatant (serum) was then transferred to a new collection tube, which was centrifuged again at 4 °C for 5 min at 5000 rpm. Hereafter, the supernatant was transferred into a DNA LoBind Eppendorf tube, which was frozen at −80 °C, prior to metabolomics analyses.

### 2.2. Metabolomic Analyses

All metabolomics analyses were performed at the Centre for Human Metabolomics (Potchefstroom, South Africa). Serum samples were analysed in a randomized order, using the commercial MxP Quant 500 kit (Biocrates Life Science AG, Innsbruck, Austria, https://biocrates.com/mxp-quant-500-kit/ (accessed on 13 October 2023), on a 5500 QTRAP^®^ instrument coupled to a UPLC (AB Sciex, Darmstadt, Germany).

This comprehensive, targeted assay was developed to detect and quantify up to 630 metabolites, from 26 biochemical classes, using two MS methods. It is important to mention that the kit, including all consumables, reagents, methods, and instructions were implemented as intended, without any deviations. The kit provides instructions and exact settings for liquid chromatography (LC) and flow injection analysis (FIA) MS/MS, operating in both positive and negative ionization modes, including instrument-specific acquisition and quantification methods. Prior to analysis, wash solvents and mobile phases were prepared as per the manufacturer’s instructions. Serum samples were loaded onto patented 96-well filter plates, which were preloaded with internal standards, calibration, and quality control samples (all included in the kit). The workflow consisted of derivatization, extraction, and dilution, following the given instructions. During the dilution process, 150 µL of supernatant from the 96-well filter plate was mixed with 150 µL of water for LC-MS/MS analysis, while 10 µL of the supernatant was diluted with the FIA mobile phase, containing the FIA additive provided in the kit, for FIA analyses.

Alkaloids, amine oxides, amino acids, amino acid-related metabolites, bile acids, biogenic amines, carboxylic acids, cresols, fatty acids, hormones, indole derivatives, nucleobase-related metabolites, vitamins, and cofactors were detected on the MS after prior chromatography, using the MxP Quant 500 column.

The second method, consisting of FIA-MS/MS, was used to detect acylcarnitines, ceramides, cholesterol esters, diacylglycerols, dihydroceramides, glycerophospholipids, glycosylceramides, sphingolipids, triacylglycerols, and sugars.

Metabolite quantification was completed via a seven-point (LC-MS/MS) or one-point (FIA-MS/MS) calibration using isotopically labelled internal standards; or semi-quantitative, using an internal standard with similar chemical properties, depending on the metabolite as predetermined in the kit’s quantification software. Sample preparation (using 10 µL serum), instrument analyses, quality control measures and checks, and metabolite quantification were performed in accordance with the manufacturer’s instructions. Data were collected with Analyst^®^ software v1.7.1 (Sciex, Framingham, MA, USA) and analysed with Biocrates MetIDQ™ v7.13.11-DB109-Nitrogen-2850 (Biocrates Life Sciences AG, Innsbruck, Austria).

### 2.3. Statistical Analysis

All statistical analyses were performed using MetaboAnalyst 5.0 [14]. Although the MxP Quant 500 kit is said to be able to measure a total of 630 metabolites after passing the included system suitability test, a large number of these were measured at concentrations below the given limit of detection and were annotated as zero (Supplementary Material Table S1). As a filter, metabolites were removed from the dataset if they had a zero value in 50% or more of the samples in each of the experimental groups. The remainder of these observations were replaced with half of the minimum value of the specific metabolite in the original data. Metabolite classes that presented challenges in detection using this filter encompassed acylcarnitines, alkaloids, bile acids, cesols, nucleobases, sphingomyelins, and trihexosylceramides. In these classes, none of the metabolites were successfully detected, indicating a limitation of the kit in capturing these specific compounds in our analysis (Supplementary Material Table S1). After the application of this filter, only 277 metabolites were available for statistical analyses, of which the data were log-transformed and autoscaled. A one-way analysis of variance (ANOVA), using the Fisher’s least significant difference method (LSD) as post hoc analyses, was applied, and a resulting *p*-value of <0.05 deemed a metabolite as significantly different between the sample groups. In addition to this univariate measure, we also applied a multivariate, supervised regression technique: partial least squares discriminant analysis (PLS-DA). In this case, metabolites were classified as important group discriminators if a value ≥ 1 was calculated as their variable importance in the projection (VIP) parameter (average of the optimal number of components), which is a weighted sum of squares of the PLS loadings. Important metabolites identified with either the univariate or the multivariate method were considered as differential and discussed accordingly.

## 3. Results

A total of 66 females, fulfilling the set inclusion and exclusion criteria, were added to the study cohort, consisting of 22 participants in each group (controls, DRSP/20EE, and 22 DRSP/30EE users).

Of the 277 metabolites included in the final dataset, 5 had *p*-values < 0.05, including betaine, glutamine, cortisol, glycine, and choline (Table 1). The noteworthy variation in all these metabolites, according to Fisher’s LSD, was between the control and COC groups, and not amongst the two COC groups.

**Figure 1:**
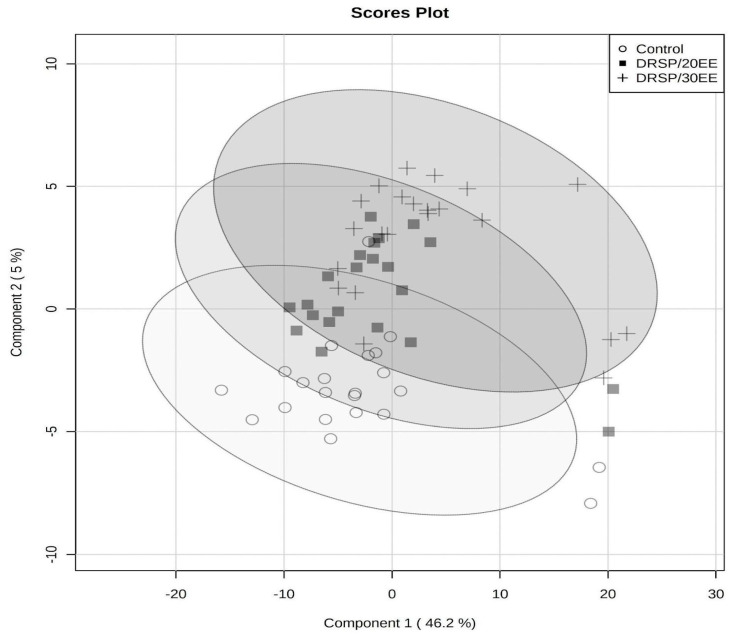
PLS-DA scores plot of the first two principal components, with the variation explained by each indicated in parentheses.

The PLS-DA model (Figure 1) validated well and was able to best classify samples into sample groups using three components (Supplementary Material Figure S1). A total of 49 compounds had VIP values ≥ 1 (Table 1), including one amine oxide, seventeen amino acids and amino acid-related metabolites, one carboxylic acid, five ceramides, one hormone-related metabolite, one indole derivative, seven phosphatidylcholines, fifteen triglycerides, and one vitamin-related metabolite. All metabolites with significant *p*-values were also classified as important variables via the PLS-DA (Table 1).

For most differential metabolites (identified as significant based on either *p*-value or VIP value criteria), the direction of the concentration change (increased or decreased) was similar for the two COC groups, with respect to the controls. However, nine differential metabolites, including mostly phosphatidylcholines and triglycerides, showed opposite changes in the two COC groups, respectively (indicated as # in Table 1).

It should be noted that, after the initial zero filtering (retaining compounds with non-zero values in 50% or more of the samples in each of the experimental groups), a significant number of metabolites still had zero values in many samples (below the given detection limit). Compounds identified as differential, which had zero values in one-third or more of the samples, across all sample groups, were interpreted with caution (indicated with * in Table 1). These included all ceramides and most triglycerides.

In total, 10 of the differential compounds were also identified as important discriminatory metabolites in our preceding studies (indicated in bold in Table 1), with the same directionality of change in the COC patient groups, compared to the controls [9,10].

## 4. Discussion

We will focus our discussion on the differential compounds which showed a similar directional change in both COC groups (compared to controls) and were detected at concentrations above the set detection limit in more than two-thirds of the total sample cohort.

Glutamine, glycine, isoleucine, leucine, proline, serine, and tyrosine corresponded to both of our previous investigations, whereas 3-methylhistidine and citrulline were discriminatory in the untargeted [9], and ornithine in the targeted study [10], respectively. These COC-induced metabolome variations suggested a phenotype corresponding to a prothrombotic state and increased oxidative stress, as discussed in these publications.

The differential compounds which were not identified as such in previous studies, include: 1-methylhistidine, 3-indole propionic acid, betaine, choline, cortisol, creatinine, hippuric acid, homoarginine, proline betaine, PC aa C38:5, PC aa C42:4, PC ae C36:4, PC ae C36:5, TG(18:0_36:2), TG(18:1_30:2), TG(18:3_36:2), TG(20:4_35:3), and trimethylamine N-oxide (TMAO).

EE reportedly induces the production of liver corticosteroid-binding globulin, consequently enhancing the ability to bind serum cortisol and raising the total cortisol concentration in COC users [15,16]. Hypercortisolism, in turn, is known to enhance haemostatic factors, specifically vWF, PAI-1, and FVIII levels while decreasing fibrinolysis activity [17,18,19,20,21,22,23]. Moreover, cortisol contributes to endothelial dysfunction [24,25] and has been implicated in reactive oxidative species (ROS) production [26]. Similarly, our results indicate a dose-dependent elevation in the cortisol levels of the COC groups compared to the controls, substantiating our previous hypothesis that COCs influence haemostatic factors directly as a means to induce a prothrombotic state and that oxidative stress plays a critical role in this process.

**Figure 2:**
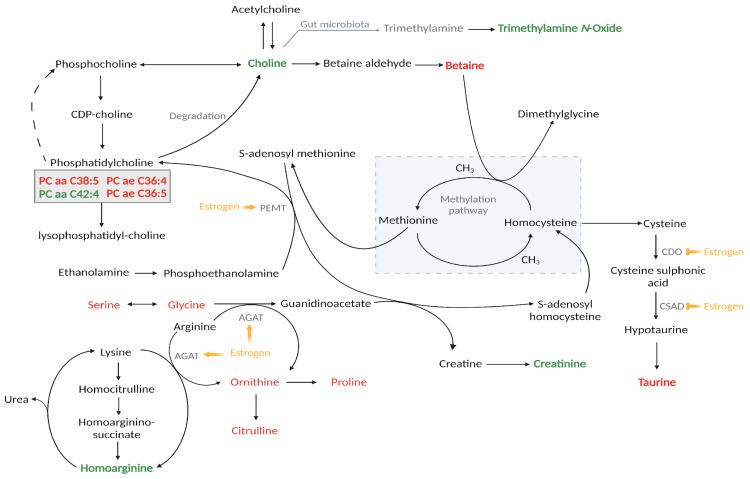
Serum metabolome interactions of the differential metabolites detected with the MxP Quant 500 kit illustrating the impact of oral contraceptive use (COC). Metabolites in green indicate elevated concentrations in both COC groups compared to controls, whereas red indicate lower concentrations in both COC groups. The known effects of estrogen on the enzymatic activity and expression of relevant enzymes are also visually represented in yellow, with induction denoted by arrows and inhibition denoted by truncated ends. AGAT, L-arginine:glycine amidinotransferase; CDO, cysteine dioxygenase; CSAD, cysteine sulfinic acid decarboxylase; PEMT, phosphatidylethanolamine N-methyltransferase. Figure created with BioRender.com (accessed on 29 September 2023).

Our results also indicate comparatively higher levels of serum homoarginine, a metabolite synthesized endogenously by L-arginine:glycine amidinotransferase (AGAT), in the COC groups compared to the controls, aligning with previous findings [27,28]. AGAT is reportedly upregulated by EE in the livers of chicks [29], which suggests that synthetic estrogen potentially also increases the expression of the related coding gene, as was seen in the presence of naturally high estrogen levels during human pregnancy [30]. Moreover, AGAT acts as a key rate-limiting enzyme in creatine synthesis [31]. Given the upregulation of AGAT, it is reasonable to expect increased creatine, and therefore also creatinine levels (Figure 2). Correlating to a previous investigation [32], elevated creatinine was indeed observed in the serum samples of both COC groups compared to those of the control group, with slightly higher concentrations detected within the higher EE dosage group (DRSP/30EE).

Vitamin J, better known as choline, is an important nutrient, particularly during pregnancy. Choline is mainly synthesised in the liver by the enzyme phosphatidylethanolamine N-methyltransferase (PEMT), although it can also be attained through dietary sources [33,34]. Choline can, in turn, be oxidised to betaine, a reaction which drives the methylation pathway (Figure 2). Previous studies have reported that choline levels exhibit a continuous increase throughout pregnancy, whereas betaine levels decreased until approximately gestational weeks 20–27, after which they plateau [35,36]. Similarly, in the context of COC use, prior research has shown reduced betaine concentrations in contraceptive users when compared to control groups [37,38], although the exact mechanisms leading to this occurrence remain unclear. In a study assessing the impact of COC use on the methylation metabolism in pre-menopausal women, significant differences in the remethylation pathway were identified between the user group and controls [38]. Specifically, and in accordance with our findings, betaine, dimethylglycine (DMG), and the betaine:choline ratio were significantly lower in the COC user group, while total cysteine, choline, and the DMG:betaine ratio were comparatively higher. During estrogen biotransformation, methylation emerges as a critical detoxification process for catechol estrogens. Since betaine plays a pivotal role as a methyl group donor in the remethylation pathway of homocysteine, the reduced betaine levels among COC users were attributed to an increased dependency on the remethylation pathway due to the higher estrogen levels [38].

Interestingly, the bacterial fermentation product of betaine and L-carnitine, TMAO, was also detected at increased concentrations in both the COC groups compared to the controls. TMAO is synthesised through enzymatic transformation of micronutrients such as choline, phosphatidylcholine, betaine, and L-carnitine by the gut microbiota, resulting in the production of trimethylamine (TMA), which is then further oxidised to form TMAO in the liver (Figure 2) [39].

The sulphur-containing amino acid, taurine, is derived from dietary sources such as dairy and meat and is also synthesised in the liver from cysteine via three enzymes: cysteine dioxygenase (CDO), cysteine sulfinic acid decarboxylase (CSAD), and hypotaurine dehydrogenase [40,41]. While investigating the impact of estrogen on taurine levels in mice, it was observed that 17-β-estradiol reduced taurine levels in both serum and cultured cells [42]. This reduction was attributed to the decreased CSAD, CDO, and taurine transporter expressions via the estrogen receptor-α [42]. In our study, we noted lower taurine concentrations in both COC groups, which could be linked to a similar mechanism to that observed in mice. It is worth noting, however, that despite our findings, several human studies have reported no changes in taurine levels following the administration of COCs [43,44,45].

A microbial byproduct derived from tryptophan, 3-indole propionic acid, holds a significant role in human health and disease [46]. In this study, we observed lower concentrations of 3-indole propionic acid in both groups of COC users, with the lowest levels detected in the higher EE dose group (DRSP/30EE). This metabolite is notable for its antioxidant and free radical scavenging properties, which are important for managing oxidative stress levels [46,47]. COC use has previously been associated with disturbances in tryptophan metabolism [47,48]. Additionally, prior research has associated reduced plasma levels of 3-indole propionic acid with disturbances in the gut microbiome [49], and several diseases [50] including human kidney disease [49] and atherosclerosis [51]. Taking into account the changes in TMAO and 3-indole propionic acid levels observed during COC use, it appears that the gut microbiome is influenced by contraceptive usage, as corroborated in previous findings [52]. However, the precise mechanisms by which these pathways are affected, and the resulting impacts, are still not completely understood.

Based on the findings above, we identified several metabolic perturbations associated with COC use. Notably, the five metabolites with significant *p*-values (betaine, glutamine, cortisol, glycine, and choline) suggest the possibility of a more pronounced metabolic disturbance within the methylation pathway.

Out of the 49 differential metabolites, a total of 40 showed a more pronounced effect in the DRSP/30EE group (marked in Table 1 with double arrows), which potentially suggests a dose-dependent effect correlating to previous studies which indicated that higher EE doses are associated with an increased risk of thrombosis [3]. However, it is important to note that the specific progestin used can influence this relationship [3]. Additionally, various factors such as age, congenital or acquired predisposition to thrombosis, the duration of COC use, and EE dosage have been linked to increased risks of stroke [53,54]. The exact metabolomic implications of different EE dosages require further investigation to be comprehensively understood and it is essential to consider these multifaceted factors when assessing the efficacy and safety of COC usage.

This is the first study to apply the commercial MxP Quant 500 kit (Biocrates Life Science AG, Innsbruck, Austria) to perform targeted, quantitative analysis and thus provide a comprehensive LC-MS/MS metabolic profile of young females using COCs. The MxP Quant 500 kit offers a notable advantage with its minimal 10 µL sample volume requirement for quantitative metabolic profiling while offering a streamlined and comprehensive workflow with all-inclusive reagents and method parameters. The similarities between the differential compounds identified in the current and previous targeted and untargeted metabolomics studies show that, although many compounds were quantified below the limit of detection using the described kit, those that were usable were indeed quantified with a high level of precision and certainty and can be used to identify biological variations between sample groups. In addition to confirming previous findings, novel observations were also made, which could lead to the elucidation of certain drug-induced mechanisms. The use of commercially available kits for LC-MS analyses could speed up method development processes and assist with the standardisation of results obtained across international laboratories and in longitudinal studies. It would, however, be advantageous if these kits could be used to analyse smaller cohorts or perhaps even individual samples, instead of the set large batch sizes, and the optimisation of individual runs for specific metabolite classes would also add value. Expanding our study to encompass more diverse cohorts could strengthen statistical validity, while longitudinal studies, specifically using a lipidomics approach, can aid in determining the long-term effects of COC use on lipid metabolism, shedding light on their safety and efficacy over time. These avenues promise to enhance our understanding of COC-use effects and their implications for women’s health and contraceptive choices.

## Figures and Tables

**Table 1:** Differential metabolites identified when considering the control and COC user groups.

			One-Way ANOVA		CONTROLS	DRSP/20EE		DRSP/30EE	
Metabolite	Metabolite Class	PLS-DA VIP (Avg Comp 1, 2, 3)	*p*-Value	Fisher’s LSD	Average Concentration (uM) (SEM)	Average Concentration (uM) (SEM)	Increased (↑) or Decreased (↓) with COC (Double Arrow Indicates Group with More Prominent Effect)	Average Concentration (uM) (SEM)	Increased (↑) or Decreased (↓) with COC
Trimethylamine N-oxide	Amine oxides	1.07	Not significant		3.714 (0.61)	4.185 (0.68)	↑	5.712 (0.98)	↑↑
**Glutamine**	Aminoacids	2.94	1.36 × 10^−6^	Control–DRSP/20EE; Control–DRSP/30EE	718.4 (27.0)	557.4 (17.0)	↓↓	564.4 (15.7)	↓
**Glycine**	Aminoacids	2.02	3.87 × 10^−5^	Control–DRSP/20EE; Control–DRSP/30EE	386.2 (23.5)	251.0 (11.8)	↓↓	298.9 (28.1)	↓
**Isoleucine**	Aminoacids	1.25	Not significant		68.55 (4.61)	54.10 (4.22)	↓	53.82 (4.84)	↓↓
**Leucine**	Aminoacids	1.03	Not significant		113.8 (6.62)	103.0 (4.49)	↓	101.8 (5.63)	↓↓
**Proline**	Aminoacids	1.02	Not significant		280.1 (27.1)	221.3 (24.0)	↓	219.3 (29.8)	↓↓
**Serine**	Aminoacids	2.05	Not significant		167.5 (7.24)	140.4 (5.72)	↓	139.5 (6.60)	↓↓
**Tyrosine**	Aminoacids	1.02	Not significant		103.1 (11.6)	72.12 (7.44)	↓↓	79.14 (11.0)	↓
1-Methylhistidine	Aminoacids related	1.47	Not significant		3.840 (0.28)	4.119 (0.19)	↑	4.621 (0.29)	↑↑
**3-Methylhistidine**	Aminoacids related	1.21	Not significant		2.653 (0.70)	2.996 (0.62)	↑	4.782 (0.95)	↑↑
β-Aminobutyric acid ^#^	Aminoacids related	1.01	Not significant		3.28 (0.36)	3.304 (0.48)	↑	2.635 (0.48)	↓
Betaine	Aminoacids related	3.47	1.60 × 10^−10^	Control–DRSP/20EE; Control–DRSP/30EE	34.18 (2.75)	14.69 (1.21)	↓↓	14.99 (1.41)	↓
**Citrulline**	Aminoacids related	1.36	Not significant		26.61 (2.33)	21.23 (1.21)	↓	19.52 (1.80)	↓↓
Creatinine	Aminoacids related	1.21	Not significant		68.88 (2.92)	71.59 (2.24)	↑	76.36 (3.08)	↑↑
Homoarginine	Aminoacids related	1.59	Not significant		1.844 (0.16)	2.383 (0.13)	↑	2.425 (0.17)	↑↑
**Ornithine**	Aminoacids related	1.76	Not significant		59.63 (5.99)	35.89 (2.07)	↓↓	36.37 (2.86)	↓
Proline betaine	Aminoacids related	1.02	Not significant		5.030 (1.40)	3.751 (1.07)	↓↓	3.915 (1.24)	↓
Taurine	Aminoacids related	1.19	Not significant		22.47 (0.91)	21.17 (1.12)	↓	20.33 (1.02)	↓↓
Hippuric acid	Carboxylic acids	1.35	Not significant		2.045 (0.36)	3.47 (0.65)	↑↑	3.007 (0.38)	↑
Cer(d18:1/22:0) *	Ceramides	1.57	Not significant		0.379 (0.05)	0.253 (0.05)	↓	0.139 (0.03)	↓↓
Cer(d18:1/23:0) *	Ceramides	2.09	1.37 × 10^−3^	Control–DRSP/30EE; DRSP/20EE–DRSP/30EE	0.821 (0.13)	0.693 (0.12)	↓	0.203 (0.08)	↓↓
Cer(d18:1/24:1) *^#^	Ceramides	1.09	Not significant		1.313 (0.30)	0.993 (0.22)	↓	1.445 (0.23)	↑
Cer(d18:0/20:0) *	Dihydro-ceramides	1.42	Not significant		0.043 (0.00)	0.039 (0.00)	↓	0.023 (0.00)	↓↓
HexCer(d18:1/22:0) *	Hexosyl-ceramides	1.21	Not significant		1.821 (0.39)	1.313 (0.35)	↓	0.636 (0.26)	↓↓
Cortisol	Hormones	2.93	1.18 × 10^−5^	DRSP/20EE–Control; DRSP/30EE–Control	0.253 (0.02)	0.405 (0.03)	↑	0.513 (0.03)	↑↑
3-Indolepropionic acid	Indoles derivatives	1.12	Not significant		0.911 (0.14)	0.803 (0.10)	↓	0.598 (0.08)	↓↓
PC aa C32:1 ^#^	Phosphatidyl-cholines	1.02	Not significant		5.270 (0.69)	5.304 (0.64)	↑	4.811 (0.72)	↓
PC aa C34:3 ^#^	Phosphatidyl-cholines	1.02	Not significant		9.674 (1.04)	9.923 (1.00)	↑	8.411 (0.95)	↓
PC aa C36:3 ^#^	Phosphatidyl-cholines	1.03	Not significant		88.06 (9.33)	96.47 (9.10)	↑	81.97 (9.09)	↓
PC aa C38:5	Phosphatidyl-cholines	1.01	Not significant		26.30 (2.63)	25.30 (2.14)	↓	22.30 (2.13)	↓↓
PC aa C42:4	Phosphatidyl-cholines	1.05	Not significant		0.158 (0.03)	0.161 (0.02)	↑	0.185 (0.02)	↑↑
PC ae C36:4	Phosphatidyl-cholines	1.00	Not significant		10.10 (0.88)	9.776 (0.88)	↓	9.251 (0.84)	↓↓
PC ae C36:5	Phosphatidyl-cholines	1.00	Not significant		7.953 (0.73)	7.401 (0.74)	↓↓	7.550 (0.71)	↓
TG(16:0_38:4) ^#^	Triglycerides	1.04	Not significant		3.145 (0.77)	3.581 (0.45)	↑	2.249 (0.33)	↓
TG(16:0_40:7) *	Triglycerides	1.09	Not significant		1.801 (0.36)	1.649 (0.31)	↓	0.763 (0.21)	↓↓
TG(18:0_36:2)	Triglycerides	1.26	Not significant		11.36 (1.81)	9.309 (1.02)	↓	5.703 (1.31)	↓↓
TG(18:1_30:2)	Triglycerides	1.19	Not significant		1.682 (0.13)	1.492 (0.12)	↓	1.184 (0.10)	↓↓
TG(18:1_34:4) *	Triglycerides	1.10	Not significant		1.647 (0.40)	1.523 (0.29)	↓	1.349 (0.21)	↓↓
TG(18:1_38:5) ^#^	Triglycerides	1.00	Not significant		2.593 (0.52)	2.679 (0.35)	↑	2.238 (0.32)	↓
TG(18:2_28:0) *	Triglycerides	1.70	Not significant		1.141 (0.21)	0.850 (0.23)	↓	0.304 (0.11)	↓↓
TG(18:2_33:1) ^#^	Triglycerides	1.01	Not significant		2.085 (0.38)	2.489 (0.37)	↑	1.747 (0.27)	↓
TG(18:2_35:3) *^#^	Triglycerides	1.09	Not significant		0.564 (0.11)	0.606 (0.09)	↑	0.262 (0.08)	↓
TG(18:2_36:5) *	Triglycerides	1.07	Not significant		5.518 (1.40)	3.08 (0.59)	↓	1.770 (0.40)	↓↓
TG(18:3_34:3) *	Triglycerides	1.09	Not significant		3.889 (1.04)	2.503 (0.65)	↓	1.200 (0.41)	↓↓
TG(18:3_36:2)	Triglycerides	1.13	Not significant		6.652 (1.26)	5.364 (0.74)	↓	2.851 (0.61)	↓↓
TG(20:4_35:3)	Triglycerides	1.01	Not significant		0.116 (0.01)	0.086 (0.01)	↓	0.078 (0.01)	↓↓
TG(22:4_32:0) *	Triglycerides	1.63	Not significant		0.154 (0.02)	0.153 (0.02)	↓	0.067 (0.02)	↓↓
TG(22:5_32:0) *	Triglycerides	1.23	Not significant		0.218 (0.05)	0.304 (0.05)	↑↑	0.274 (0.04)	↑
Choline	Vitamins & cofactors	1.98	6.33 × 10^−4^	DRSP/20EE–Control; DRSP/30EE–Control	14.10 (0.56)	18.25 (0.99)	↑↑	18.00 (0.81)	↑

* Detected below detection limit in one-third or more of samples across all sample groups. ^#^ Compounds had an opposite directional change in the two COC groups, compared to the controls. Compounds in bold were also detected as important in our previous studies, with the same directional change in the COC patients compared to the control group. Underlined compounds represent differential compounds which were not previously identified as such.

## Data Availability

Raw data were generated at the Centre for Human Metabolomics, North-West University, South Africa. Derived data supporting the findings of this study are available from the corresponding author I.d.P. on request.

## Supplementary Materials

The following supporting information can be downloaded at: https://www.mdpi.com/article/10.3390/metabo13101092/s1, Figure S1: PLS-DA classification, indicating the best classifier (red star); Table S1: Summary of the metabolites deleted from the dataset after applying a zero (below limit of detection) filter.
