# Supplementary material for: Metabolomic Profiling of Hormonal Contraceptive Use in Young Females Using a Commercially Available LC-MS/MS Kit
Source: Metabolites. 2023 Oct 18;13(10):1092. doi: 10.3390/metabo13101092 (PMC10609319; doi:10.3390/metabo13101092)
Supplement: Supplementary file 1 [file metabolites-13-01092-s001.zip › metabolites-2665567-supplementary.pdf]

Supplementary material to:

Metabolomic profiling of hormonal contraceptive use in young females using a commercially available LC-MS/MS kit

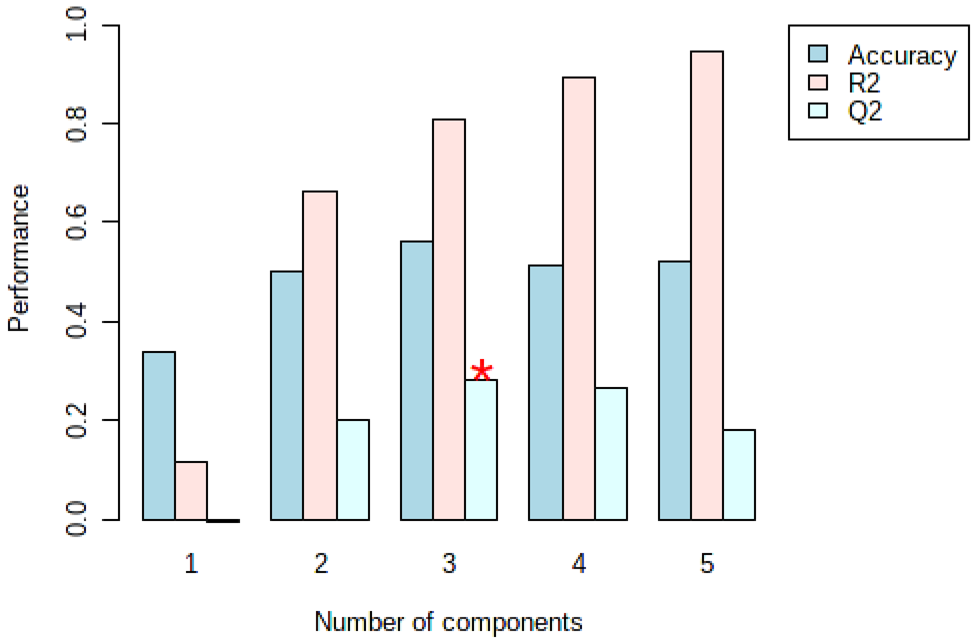

Figure S1. PLS-DA classification, indicating the best classifier (red star)

Table S1: Summary of the metabolites deleted from the dataset after applying a zero (below limit of detection) filter

**Metabolite class**	**Number of metabolites deleted**	**Total metabolites potentially detectable per class**	**% deleted**	**Used in statistical analyses**
Acylcarnitines	40	40	100	0
Alkaloids	1	1	100	0
Aminoacids	1	20	5	19
Aminoacids related	18	30	60	12
Amine oxides	0	1	0	1
Bile acids	14	14	100	0
Biogenic amines	7	9	78	2
Carbohydrates and Related	0	1	0	1
Carboxylic acids	6	7	86	1
Ceramides	24	28	86	4
Cholesterol esters	13	22	59	9
Cesols	1	1	100	0
Diglycerides	33	44	75	11
Dihexosylceramides	8	9	89	1
Dihydroceramides	7	8	88	1
Fatty acids	11	12	92	1
Hexosylceramides	16	19	84	3
Hormones	3	4	75	1
Indoles derivatives	3	4	75	1
Lysophosphatidylcholines	8	14	57	6
Nucleobases related	2	2	100	0
Phosphatidylcholines	21	76	28	55
Sphingomyelins	15	15	100	0
Triglycerides	95	242	39	147
Trihexosylceramides	6	6	100	0
Vitamins & cofactors	0	1	0	1
**TOTAL**	**353**	**630**	**56**	**277**
